# Genome-wide identification and expression analysis of *AUX/LAX* family genes in Chinese hickory (*Carya cathayensis* Sarg.) Under various abiotic stresses and grafting

**DOI:** 10.3389/fpls.2022.1060965

**Published:** 2023-01-05

**Authors:** Ying Yang, Jiayan Wang, Yan Xu, Farhat Abbas, Dongbin Xu, Shenchen Tao, Xiaoting Xie, Feng Song, Qiaoyu Huang, Anket Sharma, Luqing Zheng, Daoliang Yan, Xiaofei Wang, Bingsong Zheng, Huwei Yuan, Rongling Wu, Yi He

**Affiliations:** ^1^ State Key Laboratory of Subtropical Silviculture, Zhejiang Agriculture and Forestry (A&F) University, Hangzhou, China; ^2^ Zhejiang Provincial Key Laboratory of Forest Aromatic Plants-based Healthcare Functions, Zhejiang Agriculture and Forestry (A&F) University, Hangzhou, China; ^3^ College of Life Sciences, Nanjing Agricultural University, Nanjing, Jiangsu, China; ^4^ Departments of Public Health Sciences and Statistics, Center for Statistical Genetics, Pennsylvania State University, Hershey, PA, United States

**Keywords:** auxin, AUX/LAX, *Carya cathayensis*, drought, grafting, salt

## Abstract

Auxin is essential for regulating plant growth and development as well as the response of plants to abiotic stresses. AUX/LAX proteins are auxin influx transporters belonging to the amino acid permease family of proton-driven transporters, and are involved in the transport of indole-3-acetic acid (IAA). However, how *AUX/LAX* genes respond to abiotic stresses in Chinese hickory is less studied. For the first time identification, structural characteristics as well as gene expression analysis of the *AUX/LAX* gene family in Chinese hickory were conducted by using techniques of gene cloning and real-time fluorescent quantitative PCR. Eight *CcAUX/LAXs* were identified in Chinese hickory, all of which had the conserved structural characteristics of *AUX/LAXs*. CcAUX/LAXs were most closely related to their homologous proteins in *Populus trichocarpa* , which was in consistence with their common taxonomic character of woody trees. *CcAUX/LAXs* exhibited different expression profiles in different tissues, indicating their varying roles during growth and development. A number of light-, hormone-, and abiotic stress responsive *cis*-acting regulatory elements were detected on the promoters of *CcAUX/LAX* genes. *CcAUX/LAX* genes responded differently to drought and salt stress treatments to varying degrees. Furthermore, *CcAUX/LAX* genes exhibited complex expression changes during Chinese hickory grafting. These findings not only provide a valuable resource for further functional validation of *CcAUX/LAXs*, but also contribute to a better understanding of their potential regulatory functions during grafting and abiotic stress treatments in Chinese hickory.

## 1 Introduction

Plants experience varying degrees of biotic and abiotic stresses throughout their life cycle in the natural environment, with abiotic stresses, including drought, high salinity, high temperature, low temperature, waterlogging, and so on, being the most common environmental factors in nature, all of which affect plant natural growth and development and can even result in plant damage or death (Ramegowda and Senthil-Kumar, 2015; Pandey et al., 2017; Samadi et al., 2021; Aliyari Rad et al., 2022; Dehghanian et al., 2022). As one of the most important plant hormones, auxin affects different aspects of plant growth and development through the establishment of auxin concentration gradient which is determined by the combined actions of its synthesis, metabolism, transportation, and signal transduction. Previous studies have found that auxin regulates the normal growth of plants, including apical dominance, phototropism and geotropism, inflorescence and frond development, lateral and adventitious root formation, vascular tissue differentiation, and fruit maturity (Korver et al., 2018; Gomes and Scortecci, 2021). Furthermore, auxin is essential for the temporal coordination of plants’ responses to abiotic stresses and many environmental stress responses are dependent on the dynamic distribution of auxin in different plant tissues (Peleg and Blumwald, 2011; Rahman, 2013; Min et al., 2014; Hadi et al., 2021).

Auxin is synthesized in active parts of plants, such as shoot apex, terminal buds, young leaves, developing seeds, meristems of main root tips, and developing lateral roots (Matthes et al., 2019), and then transported to distal target tissues either through the bulk flow in stem vascular tissues in a non-polar free diffusion way or through active transport mediated by transporters (Wulf et al., 2019). Auxin transporters are considered to be involved in the active transport process of auxin. Auxin transporters mainly include AUXIN RESISTENT1/LIKE AUX1 (AUX/LAX) influx carriers, PIN-FORMED (PIN) efflux carriers, and ATP binding cassette B/P- glycoprotein/Multi-drug-resistance (ABCB/MDR/PGP) efflux/condition carriers (Hammes et al., 2022). PIN proteins are the primary auxin efflux carriers in plants, transporting auxin between or within cells (Barbosa et al., 2018; Ung et al., 2022). ABCB proteins belong to the ABC transporter protein supergene family, and the majority of which have been identified as auxin transporters that functions in the long-distance transport of auxin and the outflow of auxin in the apical tissue (Cho and Cho, 2013; Mellor et al., 2022). AUX/LAXs are multimembrane-spanning proteins involve in the uptake of auxin and the flow of auxin between cells. AUX/LAXs are related to amino acid transporters and constitute a plant-specific subclass within the amino acid/auxin permease superfamily (Péret et al., 2012; Robert et al., 2015). Numerous studies are currently being conducted on the *AUX/LAX* gene family, and the functions of certain members in some species have been identified (Swarup et al., 2008; Péret et al., 2012; Chai et al., 2016).

In *Arabidopsis*, the AUX/LAX influx carriers include four members, AUX1, LAX1, LAX2 and LAX3. Although the AUX/LAX family is highly conservative in sequences and biochemical functions, however, each member exhibits distinct temporal and spatial expression patterns and works independently or cooperatively in a variety of developmental events (Péret et al., 2012; Swarup and Péret, 2012). AUX1 was the first discovered member of the AUX/LAX family that played an important role in the root gravity response, mutation of *AUX1* result in a significant loss of root geotropism (Bennett et al., 1996). Moreover, *AUX1* was found asymmetrically in the plasma membrane of root phloem cells, where it promoted the transport of auxin from the root to the top and bottom, then advance root hair growth (Swarup et al., 2001; Marchant et al., 2002; Kleine-Vehn et al., 2006). In wild cherry (*Prunus avium* L.), *PaLAX1* accelerates the rate of auxin uptake into cells and alters the distribution of free endogenous auxin (Hoyerová et al., 2008). In *Arabidopsis*, *AtLAX3* and *AtAUX1* coordinate lateral root development by regulating the emergence and initiation of lateral root primordia (Marchant et al., 2002; Swarup et al., 2008). *LAX2* was involved in leaf vein formation and xylem development (Péret et al., 2012). The *AUX/LAX* gene family influences phyllotactic patterns and is required for the establishment of embryonic root cell organization and plant embryogenesis in *Arabidopsis* (Bainbridge et al., 2008; Ugartechea-Chirino et al., 2010; Haskovec et al., 2019). In addition to the aforementioned functions, the auxin influx carrier is involved in processes such as seed germination, adventitious roots, and female gametophyte development (Panoli et al., 2015; Chai et al., 2016; Lee et al., 2019). Furthermore, auxin transport may function in the interaction of symbiosis and pathogenic plant microorganisms, influencing auxin penetration in host plant cells (Lee et al., 2011).

There is increasing evidence that AUX/LAX auxin transporters function in plants’ adaptation to changing environmental conditions. In *Arabidopsis*, an increase in the expression of *AUX1* and auxin biosynthesis-related genes following ethylene treatment led to an increase in auxin accumulation, thereby regulating the root growth inhibition mediated by alkali stress (Li et al., 2015). *AtAUX1* played an important role in arsenic-induced oxidative stress tolerance (Krishnamurthy and Rathinasabapathi, 2013). *OsPIN3* was involved in auxin transport and drought stress response in rice, indicating that the polar auxin transport pathway is involved in regulating plant response to adversity stress (Zhang et al., 2012). Furthermore, drought stress altered the transcriptional expression profiles of *AUX/LAX* genes. It was found that five *OsAUX/LAXs* responded to drought and salinity, with most *OsLAXs* down-regulated and three up-regulated in some rice tissues and under drought, salt, and abscisic acid (ABA) treatments (Chai and Subudhi, 2016). Due to drought stress, in maize (*Zea mays* L.), most *ZmLAX* genes were up-regulated in stems but down-regulated in roots, implying dynamic growth hormone transport between stems and roots under abiotic stress (Yue et al., 2015). In soybean (*Glycine max* L.), most *GmLAXs* were down-regulated by drought and dehydration, while only six *GmLAXs* were down-regulated by salt stress (Chai et al., 2016). In *Boehmeria nivea* L., the *BnAUX/LAXs* responded to drought stress at the transcriptional level, with differential expression in leaves and roots (Bao et al., 2019). However, sorghum (*Sorghum bicolor* L.) *LAXs* exhibited irregular expression patterns under drought and salt stress (Chai et al., 2016). The above evidences reveal the responses of *AUX/LAX*s to abiotic stress, which provides a foundation for further investigation of the function of *AUX/LAX* genes.

Chinese hickory (*Carya cathayensis* Sarg.) is a commercially important tree species that produces nuts with a high nutritional value and has thus become one of the economic pillars for mountain farmers seeking to escape poverty (Cao et al., 2012; Ni and Shi, 2014; Yuan et al., 2018). However, during growth and development, Chinese hickory is vulnerable to environmental hazards such as drought, salinity, and high temperatures, resulting in slow growth, leaf loss, flower and fruit drop, and even tree death, resulting in reduced fruit quality and yield (Xi et al., 2016; Sharma et al., 2020; Zhang et al., 2021; Jiao et al., 2022). Grafting is widely used to improve plant adaptation to biotic or abiotic stresses (Assunção et al., 2019). In recent years, grafting has been regarded as one of the most promising techniques to solve the problems of Chinese hickory, including big tree size, sensitive to biotic and abiotic stresses, and long juvenile phase. However, the survival rate of grafting in Chinese hickory are still low because of the complex factors influencing the whole grafting process, which has restricted the development of Chinese hickory industry to a large extent. Consequently, revealing the regulatory mechanisms of grafting success would be of great importance to promote the development of Chinese hickory. The adhesion between rootstock and scion is the first step in successful grafting, followed by the formation of the cambium, and finally, the establishment of vascular connections (Kurotani and Notaguchi, 2021). The content of auxin in the healing area increased significantly after grafting and remained high levels throughout the healing process (Sharma and Zheng, 2019). Several studies about Chinese hickory grafting have identified the key stages important for survival (Liu et al., 2002), and have concluded that auxin plays an important role during the grafting success (Zheng et al., 2002; Qiu et al., 2016; Xu et al., 2017; Saravana Kumar et al., 2018; Yuan et al., 2018; Xu et al., 2020; Yang et al., 2021). Despite the significant advances in the studies of Chinese hickory and Chinese hickory grafting, little is known about the genetic roles of key genes, including auxin influx carriers under abiotic stress treatments and during grafting.

In the current study, the *AUX/LAX* family genes in Chinese hickory were cloned and structurally analyzed in terms of phylogeny analysis, gene structure analysis, and detection of *cis*-acting regulatory elements on their promoters. We explored the expression patterns of the *CcAUX/LAX* genes in response to salt, drought, and grafting treatments. The tissue-specific expression profiles of *CcAUX/LAX* genes, as well as their differential responses to salt and drought stress, provide the molecular basis for improving abiotic stress tolerance in Chinese hickory. Simultaneously, our study provided new insights into the expression of the *CcAUX/LAX* gene family during the grafting process and investigated the transport of auxin during the grafting mechanism, laying the groundwork for future research into the mechanism of auxin in the graft healing process.

## 2 Materials and methods

### 2.1 Plant materials, growth conditions and treatments

Seeds of Chinese hickory were collected in September, 2015, and infiltrated in carbendazim solution for about 1 hour. After infiltration, seeds were spread on the mixed medium containing 55% medium (Hangzhou Jinhai Agricultural Technology Co., LTD) and 45% yellow sandy soil, and covered with the same mixed medium for the thickness of about 5 cm. The seeds were then cultured for about 4 months at the temperature of ~28°C and humidity of 70%~80% until germination. After germination, the seeds were cultured on the seedbeds containing 10 cm river sand and 5 cm mixed medium (containing 40% peat soil, 30% wormcast and 30% silt soil) at the following conditions: temperature ~15°C, humidity 60~75%. The seedlings were watered every 4 days and disinfected every 20 days until reaching the height of ~10 cm. The seedlings were then transplanted into the cups with the size of 17 cm × 22 cm containing the medium 50% peat soil, 30% pastoral soil, 10% organic fertilizer, 5% epicarp, 3% perlite and 2% slow release fertilizer in May 2016. One seedling were planted in each cup and the seedlings were cultured in the greenhouse of Zhejiang Agricultural and Forestry University until April 2018 (two years old) at the following conditions: temperature 25~35°C, humidity 70%, photoperiod 12/12-hour light/dark. As a control for the abiotic stress treatment, Chinese hickory seedlings were irrigated with tap water. Chinese hickory seedlings were irrigated with 50 g·L^-1^ and 200 g·L^-1^ PEG6000 (polyethylene glycol) solutions until thoroughly watered, repeated every 3 days, and samples were collected 5 days, 10 days, and 15 days after treatment, respectively, under simulated drought stress. For salt stress treatment, the Chinese hickory seedlings were thoroughly watered with 50 mM and 150 mM NaCl solutions, repeated every 3 days, and samples were collected after 1 day, 3 days, and 10 days of treatment. The roots, stems, and leaves of Chinese hickory seedlings were collected at various stress levels and time intervals. For each tissue, collections from five different seedlings were mixed together and regarded as a single sample. For the tissue-specific expression analysis, we collected the tissues of Chinese hickory, stems, leaves, flowers and fruits. For each tissue, collections from five different seedlings were mixed together and regarded as a single sample. The grafting samples were the same as a previous work of our group (Xu et al., 2020). Graft unions of four replicates in each group were used for expression analysis of *CcAUX/LAX* genes. The samples were immediately immersed in liquid nitrogen and stored in a -80°C ultra-low temperature refrigerator.

### 2.2 Isolation and cloning of *AUX/LAX* genes in Chinese hickory

The sequences of *AUX/LAX* family genes in Chinese hickory were screened from previously published genome sequences (http://dx.doi.org/10.5524/100571) (Huang et al., 2019). The BLASTX algorithm was used to identify all unigenes, and overlapping sequences were assembled using the SeqMan software in the Lasergene software package. The eight assembled sequences were analyzed and found to be full-length *AUX/LAX* gene sequences from Chinese hickory. After identifying the full-length gene sequences, the genes were amplified with gene-specific primers (**Supplementary Table 1**) using the PrimeSTAR Max DNA Polymerase (TaKaRa, Dalian, China).

### 2.3 Phylogeny, gene structure, and MEME analysis

ClustalW was used to perform multiple sequence alignments of AUX/LAX proteins from *Arabidopsis thaliana*, *Populus trichocarpa*, *Oryza sativa*, *Vitis vinifera*, *Eucalyptus grandis* and *Prunus persica*. The phylogenetic tree was constructed using MEGA-X (version 10.0.1) with the neighbor-joining (N-J) method (1000 bootstrap replicates). To analyze gene structures, the Gene Structure Display Server (GSDS) online program (http://gsds.cbi.pku.edu.cn/) was used to compare the coding sequences of the *CcAUX/LAX* family genes with their corresponding genome sequences. TMHMM2 was used to analyze and visualize the transmembrane domains of CcAUX/LAXs. Protein secondary structure prediction with SOPMA (https://npsa-prabi.ibcp.fr/cgi-bin/npsa_automat.pl? page=npsa_sopma. html). ExPASy ProtParam (http://www.expasy.org/proteomics) was used to analyze the physiochemical parameters of CcAUX/LAXs, such as protein length, molecular weight (MW), and isoelectric point (pI). The Multiple Expectation Maximization for Motif Elicitation (MEME) Suite (https://meme-suite.org/tools/meme) was used to analyze the motifs of CcAUX/LAX family proteins, with the number of motifs set to eight (Bailey et al., 2009).

### 2.4 Analysis of cis−elements in CcAUX/LAX promoter region

The Chinese hickory Genome Database was used to search for the promoter sequence of the *CcAUX/LAX* genes based on the ORF sequence. For promoter analysis, the 2000 bp upstream the start codon (ATG) of *CcAUX/LAX* genes were retrieved from the Chinese hickory genome dataset and submitted to the PlantCARE database (Lescot et al., 2002) and affirmed by employing PLACE databases (Higo et al., 1999).

### 2.5 Subcellular localization analysis

The full-length coding sequences of *CcLAX1*, *CcLAX2* and *CcLAX4* were identified in Chinese hickory and constructed into the pCAMBIA1300 vector, which contains the CaMV 35S promoter and GFP gene, resulting in a 3*5S:: CcLAX: GFP* fusion vector. The plasmid was then transformed into *Agrobacterium* strain GV3101 competent cells (WEIDI, AC1001). pm-rk was used as a plasma membrane marker. All strains were transformed into 5~6-week-old tobacco (*Nicotiana benthamiana*) leaves. After 48 hours of incubation in the dark, fluorescence images were acquired at 488 and 594 nm with a high-resolution laser confocal microscope (LSM880, Zeiss).

### 2.6 RNA isolation and real-time quantitative PCR analysis

Total RNA was isolated using the MiniBEST Universal RNA Extraction Kit (TaKaRa Bio, Code No. 9767) as specified by the manufacturer. The first-strand cDNA synthesis was carried out in accordance with the manufacturer’s instructions using a PrimeScript RT Master Mix (TaKaRa Bio, Code No. RR036A). The cDNA used for Real-Time Quantitative PCR analysis (qRT-PCR) was obtained by PrimeScriptTM RT Master Mix (TaKaRa Bio, Code No. RR036A). The qRT-PCR primer sequence is listed in **Supplementary Table 2**. The qRT-PCR experiments were carried out on a BioRad CFX96 real-time PCR instrument with TB Green Premix Ex Taq II (TaKaRa Bio, Code No. RR420A). The Chinese hickory *ACTIN* gene (*CcActin*) was used as a reference gene. All the expression analyses were repeated four times. Heat map was created by MeV software using average Ct values to show expression data.

### 2.7 Statistical analysis

The 2^−ΔΔCT^ method described by Schmittgen and Livak (2008) was used to calculate relative gene expression levels. (Schmittgen and Livak, 2008). To describe the differences in expression of each CcAUX/LAX among different tissues, a one-way analysis of variance (ANOVA) was performed using IBM SPSS statistical software (version 22.0), and multiple comparisons were made using Duncan’s method at the 0.05 level of significance. The experiments were performed in three independent biological replicates.

## 3 Results

### 3.1 Identification of *AUX/LAX* family genes in Chinese hickory

The open reading frame (ORF) sequences of eight *CcAUX/LAX* genes were screened and identified based on Chinese hickory genome and transcriptome data (Qiu et al., 2016; Huang et al., 2019). To validate the accuracy of sequences, the full-length cDNA sequences of 8 *CcAUX/LAX* genes were cloned from Chinese hickory. The ORF of the *CcAUX/LAX* genes ranged from 1380 (*CcLAX5*) to 1479 (*CcLAX3*), and the number of corresponding amino acids varies from 459 aa to 494 aa (**Table 1**). The molecular weight of the proteins ranged from 52.20 kDa to 55.75 kDa, and the predicted isoelectric points varied from 8.29 (CcLAX6) to 9.01 (CcLAX8).

**Table 1:** The physical and chemical properties of AUX/LAX family proteins in Chinese hickory.

Gene	Locus ID	ORF (bp)	Length (aa)	Exon Number	Mol wt (Da)	pI	No. of transme mbrane	Predicted Location (s)
*CcLAX1*	CCA0973S0065	1440	479	8	53911.97	8.59	10	Cell membrane
*CcLAX2*	CCA0680S0089	1425	474	8	53593.60	8,30	10	Cell membrane
*CcLAX3*	CCA0748S0001	1479	494	8	55750.35	8.83	10	Cell membrane
*CcLAX4*	CCA0803S0065	1398	464	8	52497.51	8.86	10	Cell membrane
*CcLAX5*	CCA0587S0115	1380	459	7	52207.3	8.46	10	Cell membrane
*CcLAX6*	CCA0784S0024	1425	474	8	53480.41	8.29	10	Cell membrane
*CcLAX7*	CCA0522S0016	1464	487	8	54907.29	8.93	10	Cell membrane
*CcLAX8*	CCA0803S0065	1398	465	8	52681.80	9.01	10	Cell membra ne

pI means isoelectric point. Mol wt means molecular weight.

Prediction of the CcAUX/LAX transporter’s transmembrane structure revealed that all members of the family had 10 transmembrane structures (**Supplementary Figure 1**). The ratio of the secondary structure components of CcAUX/LAXs were analyzed by SOPMA, and the secondary structure composition of the eight proteins was relatively similar, with more random coils and α-helices (74.07%-78.7%), and less β-turns and extended chains (**Table 2**; **Supplementary Figure 2**).

**Table 2:** Protein secondary structure of AUX/LAX protein in Chinese hickory.

Protein	Alpha helix	Beta turn	Extended strand	Random coil
CcLAX1	46.97%	3.76%	17.54%	31.73%
CcLAX2	46.30%	3.59%	18.60%	31.50%
CcLAX3	42.86%	3.88%	20.00%	33.27%
CcLAX4	45.04%	5.17%	19.40%	30.39%
CcLAX5	42.48%	3.49%	22.44%	31.59%
CcLAX6	45.99%	4.01%	20.46%	29.54%
CcLAX7	44.44%	3.70%	19.75%	32.10%
CcLAX8	43.87%	3.66%	19.14%	33.33%

### 3.2 Phylogenetic analysis of CcAUX/LAX family members

**Figure 1:**
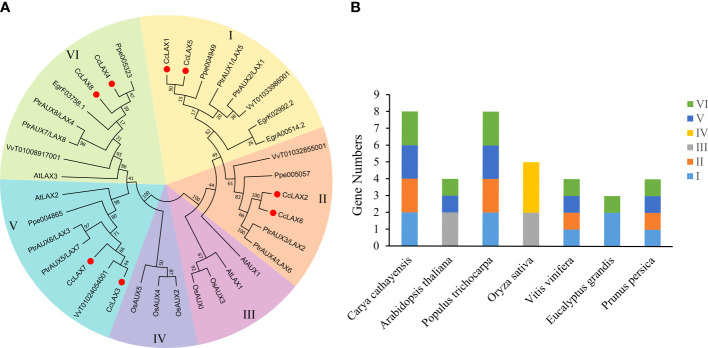
Phylogenetic analysis of *CcAUX/LAX* family genes with other *AUX/LAX* genes from 6 species. **(A)** The phylogenetic tree.The unrooted phylogenetic tree was built in MEGA6 using the neighbor-joining (N-J) method, and the bootstrap values were calculated using 1000 iterations. Different colored backgrounds indicate different groups. **(B)** The number and distribution of AUX/LAXs in different species. At, *Arabidopsis thaliana*; Pt, *Populus trichocarpa*; Os, *Oryza sativa*; Vv, *Vitis vinifera*; Eg, *Eucalyptus grandis*; Pp, *Prunus persica*.

To better understand the evolutionary relationships of *CcAUX/LAX* genes with their homologous genes in other plant species, the protein sequences of AUX/LAXs in Chinese hickory and the other 6 species, including *Arabidopsis thaliana*, *Populus trichocarpa*, *Oryza sativa*, *Vitis vinifera*, *Eucalyptus grandis* and *Prunus persica* were compared on a phylogenetic tree, detailed information on the 36 AUX/LAX proteins is shown in **Supplementary Table 3**, which may help to predict the potential functions of *CcAUX/LAXs*. The 36 AUX/LAX proteins from seven different plant species were classified into Group I, II, III, IV, V and VI (**Figure 1**), with the number of members of 8, 6, 4, 3, 7 and 8, respectively. CcAUX/LAXs were mainly distributed in four groups of I, II, V and VI. There are 3 paralogous gene pairs in the Chinese hickory *AUX/LAX* family: *CcLAX4/CcLAX8*, *CcLAX2/CcLAX6* and *CcLAX1/CcLAX5*. Furthermore, CcAUX/LAXs were found to be more closely related to the homologs in the four woody plants, but less related with the homologs in *A. thaliana* and *O. sativa* which were mainly distributed in groups III and IV. The phylogenetic relationships between AUX/LAXs suggested that they might perform different functions on the growth and development of different plant species.

### 3.3 Motif and gene structure analysis of the *CcAUX/LAX g*ene*s*

**Figure 2:**
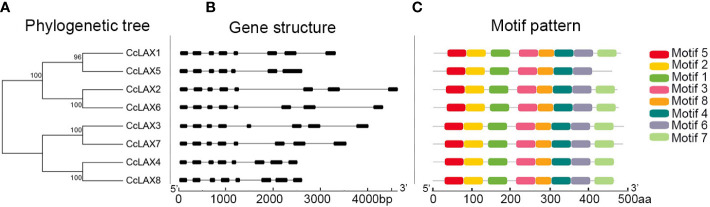
Phylogenetic analysis, gene structure and motif pattern in *AUX/LAX* genes from Chinese hickory. **(A)** The phylogenetic tree was constructed based on the full-length sequences of CcAUX/LAX proteins using MEGA7 software. **(B)** The exons are represented by black boxes, and the introns are represented by lines. **(C)** Motifs of the AUX/LAX proteins were analyzed by the Multiple Expectation Maximization for Motif Elicitation (MEME) web server, different colored boxes mean different motifs.

To understand the possible structural evolution of CcAUX/LAXs, the conserved motifs of the AUX/LAX sequences were evaluated using the MEME Suite web program. The results revealed that the majority of CcAUX/LAXs contained eight motifs, but motif 7 was missing for CcLAX5 (**Figures 2A, B**; **Supplementary Figure 2**). The structures of the *CcAUX/LAX* genes were further evaluated, and the variation of the intron-exon structures of the *CcAUX/LAX* was analyzed using GSDS online software. The results revealed that the gene structure differences among members of this family were relatively negligible, and their exon lengths were comparable. *CcLAX5* had 7 exons and 6 introns, while the other genes have 8 exons and 7 introns (**Figure 2C**). These results indicated that CcAUX/LAXs had highly conservative characteristics during evolution.

### 3.4 Cis−acting regulatory elements in the promoters of *CcAUX/LAXs*

**Figure 3:**
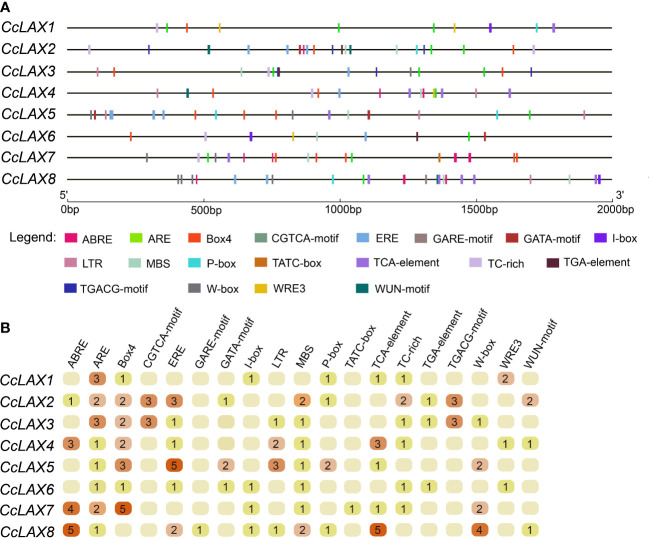
The *cis*-element prediction of *CcAUX/LAX* promoters. **(A)** The distribution of the 12 *cis*-acting elements in the 2000 bp promoter region is shown. The different types of *cis*-acting elements are represented by different shapes. ABRE, *cis*-acting element involved in the abscisic acid response. CGTCA-motif, MeJA-response element. ERE motif, *cis*-acting response involved in the ethylene response. P-box and GARE-motif, elements involved in the gibberellin response. TCA-element, *cis*-acting element associated with the salicylic acid response. TGA element, *cis*-acting element involved in the auxin response. CCAAT-box, abiotic stress response elements. LTR, *cis*-acting element involved in the low temperature response, MBS, MYB binding site involved in drought-inducibility. TC-rich, involved in defense and abiotic stress. W-box, WRKY binding site. **(B)** Number of cis-acting elements on the promoters of different CcAUX/LAXs.

The *cis*-regulatory elements upstream the 2000 bp of the start codon of the *CcAUX/LAX* genes were analyzed using PlantCARE online software. Aside from promoter core elements like the TATA-box and CAAT-box, several light-responsive elements, hormone-responsive elements, and abiotic stress-responsive elements were detected. The hormone and stress-related responsive elements were shown in **Figure 3**. The hormone responsive elements included ABRE (ACGTG, abscisic acid response), CGTCA-motif (CGTCA, methyl jasmonate response), ERE-motif (ATTTTAAA, ethylene response), P-box (CCTTTTG, gibberellin response), GARE-motif (TCTGTTG, gibberellin response), TCA-element (CCATCTTTTT, salicylic acid response) and TGA-element (AACGAC, auxin response), while abiotic stress response elements included CCAAT-box (CAACGG, MYB binding site), LTR (CCGAAA, involved in low temperature response), MBS (CAACTG, involved in drought response), TC-rich repeats (GTTTTCTTAC, involved in defense and abiotic stress) and W-box (TTGACC, WRKY binding site).

### 3.5 Tissue−specific expression profiles and subcellular localization of *CcAUX/LAX* genes

**Figure 4:**
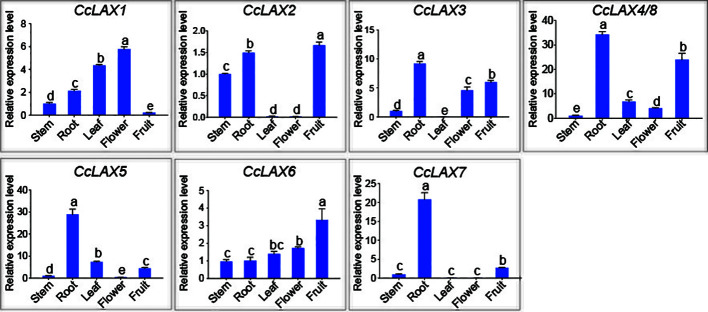
Expression patterns of *CcAUX/LAX* genes in Chinese hickory tissues (stem, root, leaf, flower, fruit). The *CcActin* gene was used as the internal reference gene for normalization. The relative expression levels of each *CcAUX/LAX* gene in stems were standardized as 1. Different letters represent a significant level of expression (*P* < 0.05) in different tissues of Chinese hickory. Multiple comparison test was performed by one-way analysis of variance and Duncan test.

**Figure 5:**
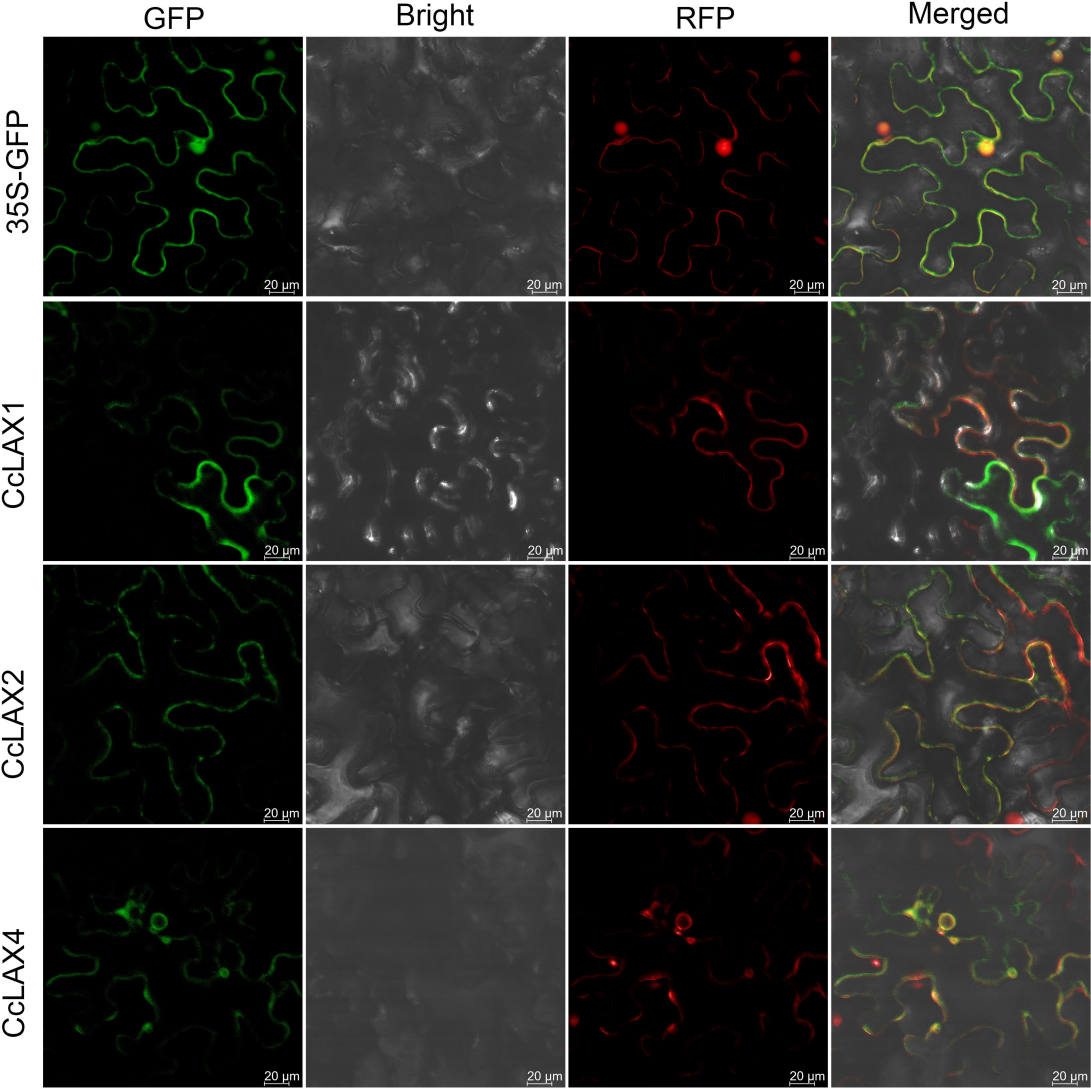
Subcellular localization of CcLAX1, CcLAX2 and CcLAX4 proteins. Left to right: green fluorescence (GFP), bright field (Bright), red fluorescence (RFP), and merged microscope images (Merged). Bar = 20μm.

To examine the potential roles of the *AUX/LAX* genes in different tissues of Chinese hickory such as roots, stems, leaves, flowers, and fruits, RT-qPCR assay was performed (**Figure 4**). Since the *CcLAX4/8* sequences were highly conservative, it is difficult to design specific expression primers to distinguish them from each other, the expression levels of these genes were the combined expression of two gene copies. The results showed that *CcLAX1* had the highest expression in flowers and lowest expression in fruits compared to other tissues, while expression of *CcLAX2* was higher in roots and fruits, but almost undetectable in leaves and flowers. Expression of *CcLAX6* was un-significantly changed between roots, stems, leaves, and flowers, but was the highest in fruits. *CcLAX5*, *CcLAX3*, *CcLAX7*, and *CcLAX8* were expressed more abundantly in roots than in other tissues. These finding suggested that the *CcAUX/LAX* genes expressed differently in different tissues, implying that they might play different roles in the growth and development stages of Chinese hickory. The subcellular localization detected in tobacco leaf epidermal cells indicated that CcLAX1, CcLAX2 and CcLAX4 were membrane-localized (**Figure 5**).

### 3.6 Expression analysis of *CcAUX/LAXs* under drought and salt stresses

**Figure 6:**
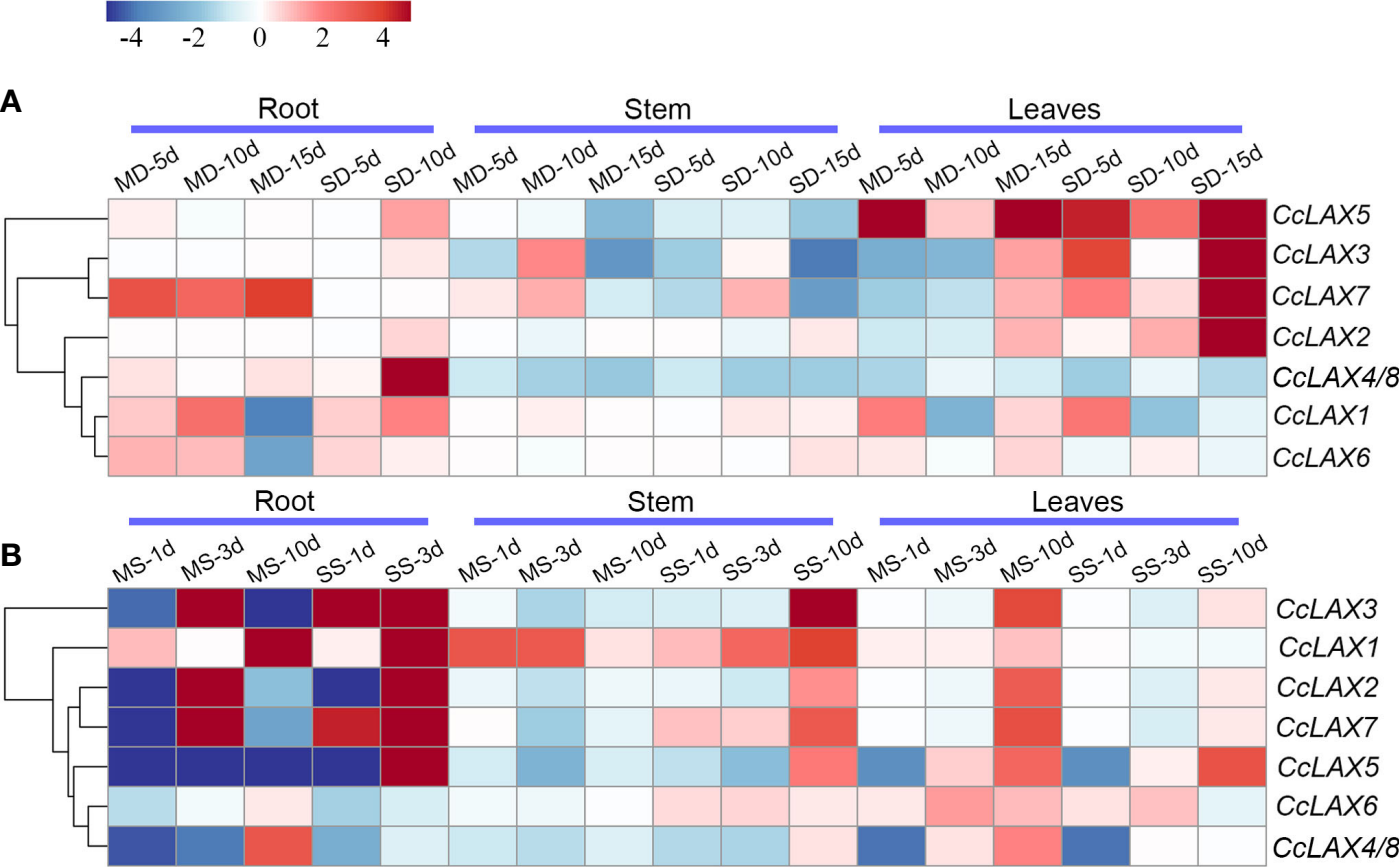
Expression patterns of putative *CcAUX/LAX* genes in Chinese hickory under drought and salt treatments. **(A)** Expression profiles of *CcAUX/LAX* family genes in roots, stems and leaves under mild drought stress (50g·L^-1^ PEG) and severe drought stress (200g·L^-1^ PEG) treatments for 5 days, 10 days and 15 days, respectively **(B)** Expression profiles of *CcAUX/LAX* family genes in roots, stems and leaves under mild salt stress (50 mM NaCl) and severe salt stress (150 mM NaCl) treatments for 1 days, 3 days and 10 days, respectively. MD-5d, mild drought stress treatment for 5 days; MD-10d, mild drought stress treatment for 10 days; MD-15d, mild drought stress treatment for 15 days; MS-1d, mild salt stress treatment for 1 days; MS-3d, mild salt stress treatment for 3 days; MS-10d, mild salt stress treatment for 10 days.

Auxin has been implicated in stress responses in numerous studies, and a significant number of auxin transporter genes have been linked to abiotic stress responses. To explore the potential function of *CcAUX/LAX* genes under environmental stress, qRT-PCR was used to assess the expression patterns of *CcAUX/LAX* genes in Chinese hickory roots, stems, and leaves after mild and severe salt stress and drought stress treatments (**Figure 6**). The expressions of *CcAUX/LAXs* were altered to varying degrees in the current study after drought and salt treatments. Under mild and severe drought stress, the expressions of some genes in roots were lower than those in leaves. For instance, the expressions of *CcLAX5*, *CcLAX3* and *CcLAX2* were down-regulated in roots, whereas increased in leaves (**Figure 6A**). After mild drought stress treatment, the expression level of *CcLAX2* in roots was greatly increased, while no significant difference was observed under severe drought stress. At the early stage of drought treatment, *CcLAX6* expression was up-regulated in roots, but there was no obvious change in leaves and stem segments. Furthermore, there was no statistically significant difference in the expression of the *CcLAX4* and *CcLAX8* genes after drought stress treatment.

Under salt stress treatment, most of the genes were highly expressed in roots, but low expression was found in stems and leaves (**Figure 6B**). The results showed that the expression levels of *CcLAX1*, *CcLAX2*, *CcLAX3*, *CcLAX5* and *CcLAX7* in roots were the highest after 3 days of severe salt stress treatment. *CcLAX3* was up-regulated in roots after both mild and severe salt stress treatments. The expression of *CcLAX6* was down-regulated in roots and up-regulated in leaves after salt stress treatment. Interestingly, a few genes had higher levels of expression after drought stress application, whilst their transcript levels were decreased under salt stress treatment. Likewise, few had higher expression levels under salt stress, while low expression was found under drought stress, indicating their different roles under different circumstances.

### 3.7 Expression profiles of *CcAUX/LAX* genes during grafting

**Figure 7:**
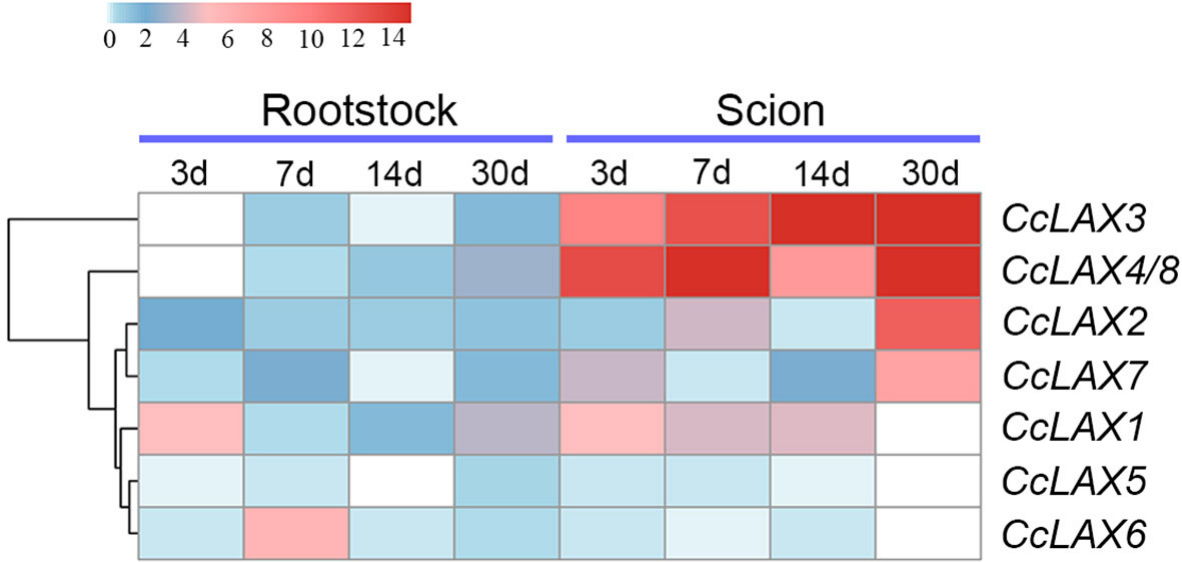
Heatmap of *CcAUX/LAX* gene expressions at different grafting stages. Red and blue represent relatively high and low expression compared to the control, respectively. The values used in the heat map were the mean of three replicates, and normalized to the control sample. The control samples were scions and rootstocks collected at day 0 of grafting.

According to our previous study, we found that the critical period for grafting healing of Chinese hickory was 3, 7, and 14 days after grafting respectively (2020; Qiu et al., 2016; Xu et al., 2017; Saravana Kumar et al., 2018). 30 days after grafting was selected to represent reconnection between the rootstock and the scion (Xu et al., 2020). To understand the possible role of *CcAUX/LAXs* in the grafting process of Chinese hickory, the expression patterns of *CcAUX/LAXs* at different time points after grafting in scions and rootstocks were analyzed. The results revealed that *CcAUX/LAX* genes had different expression patterns during different stages after grafting (**Figure 7**). Interestingly, a few genes showed differential expression patterns during the grafting process. The expression levels of *CcLAX5* and *CcLAX6* were reduced in both rootstocks and scions after grafting, but the reduction was not significant. Most genes were expressed at a higher level in the scion than in the rootstock. *CcLAX1*, *CcLAX3*, *CcLAX4* and *CcLAX8* were highly expressed in the scion and had higher levels of expression at 3, 7, 14, and 30 days after grafting, implying that they might play crucial role during grafting healing.

**Figure 8:**
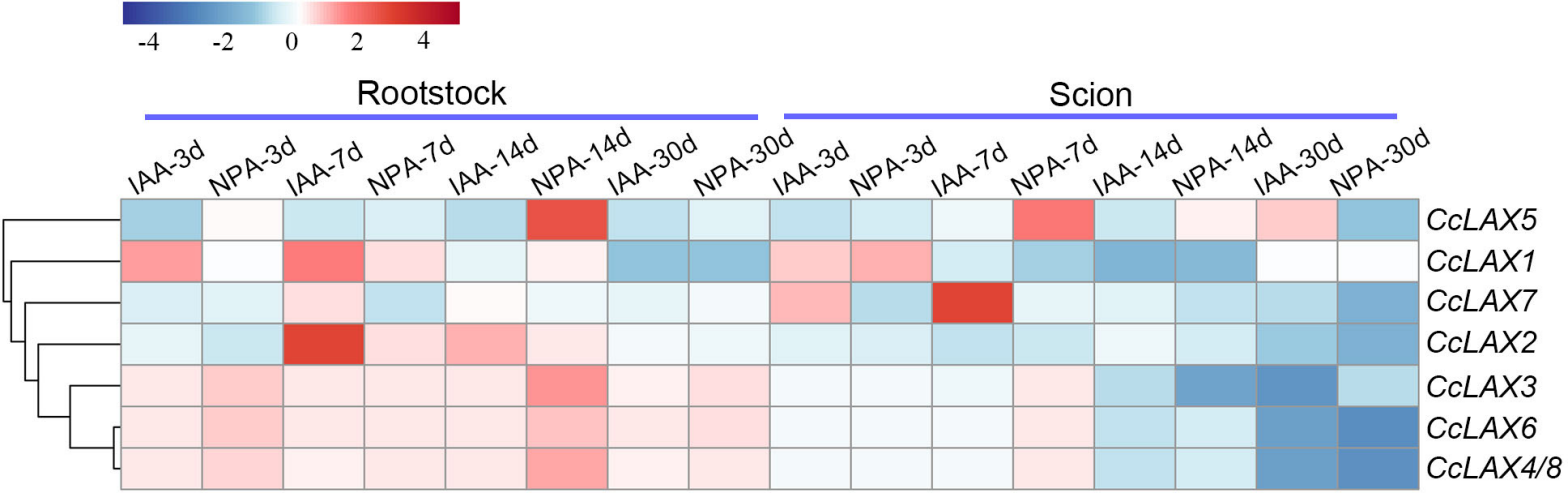
Expression patterns of *CcAUX/LAX* genes in response to IAA and NPA during grafting. Red and blue represent relatively high and low expression compared to the control, respectively. The values used in the heat map were the mean of four replicates, and normalized to the control sample. The control samples were scion and rootstock collected at day 0 of grafting.

IAA (indole-3-acetic acid) is an important plant hormone that regulates various aspects of plant growth and development, NPA (N-1-naphthylphthalic acid) is an inhibitor that inhibits or blocks the polar transport of auxin. A previous study found that IAA application significantly increased the rate of successful grafting (80%), whereas NPA application decreased the rate of successful grafting (24%), when compared to non-applied samples (32%) in Chinese hickory (Saravana Kumar et al., 2018). The expression patterns of the *CcAUX/LAX* genes under IAA and NPA treatments were examined to further reveal their potential role in the grafting process (**Figure 8**). After IAA treatment, the expression of *CcLAX2* increased in rootstocks at 7 days and 14 days after grafting, and tended to be stable at 30 days after grafting. In contrast, in the scion, the expression of *CcLAX2* gene was down-regulated. However, the expression pattern of *CcLAX5* was opposite to *CcLAX2*, with decreased expression in rootstocks after IAA treatment. The expression levels of *CcLAX3*, *CcLAX6* and *CcLAX4/8* genes were up-regulated in rootstocks after NPA and IAA treatment, and the up-regulation of gene expression was greater at 3 and 14 days after NPA treatment. In the scions, hormone treatment did not affect the expression of *CcLAX3*, *CcLAX6* and *CcLAX4/8* genes in the pre-grafting period, while gene expression was down-regulated in the post-grafting periods. It was also observed that the expression levels of most genes tended to be stable after 30 days of grafting. In addition, different genes had great differences in the degree of responses to IAA and NPA treatments.

## 4 Discussion

Auxin plays an active role in many plant developmental processes, as well as in coordinating plant responses to biotic and abiotic stresses (Petrášek and Friml, 2009; Swarup and Péret, 2012; Kazan, 2013; Sharma and Zheng, 2019). AUX/LAX is an auxin influx transporter protein, which is involved in the establishment of auxin concentration gradient and the regulation of plant grafting and abiotic stress. In this study, eight *CcAUX/LAX* genes in Chinese hickory were identified according to the published genome. And the expression profiles were determined during drought and salt stress as well as grafting to elucidate the function of CcAUX/LAXs.

### 4.1 Characterization analysis of *CcAUX/LAX* genes in Chinese hickory

Characterization analysis of *CcAUX/LAX* genes (8 members) in Chinese hickory showed that the number of *LAX* genes in Chinese hickory was consistent with that in *P. trichocarpa*, but higher than *Arabidopsis* (4 members), *O. sativa* (5 members), *V. vinifera* (3 members) and *P. persica* (5 members). Phylogenetic analysis of 36 AUX/LAX proteins in 6 plants species was carried out to compare with CcAUX/LAXs. On the phylogenetic tree, CcAUX/LAX were close to PtLAX, indicating that they may come from a common ancestor. Moreover, the result showed that three paralogous homologous gene pairs (*CcLAX4/CcLAX8*, *CcLAX2/CcLAX6* and *CcLAX1/CcLAX5*) were found in the *CcAUX/LAX* gene family (**Figure 1**), which was the same as poplars (Carraro et al., 2012). These results suggested that the expanded *CcAUX/LAX* gene family may be due to genome-wide replication during the evolution of woody plants. The protein similarity and physicochemical properties showed that the protein similarity of CcAUX/LAX family reached 72.12%-94.08% (**Supplementary Table 4**), with the similar physicochemical properties. The gene structure of *CcAUX/LAXs* exhibited a much conserved exon-intron organization with eight exons and seven introns excepted the *CcLAX5*, which was similar to the gene structures of AUX/LAXs in other plants, with 6-8 exons in *Arabidopsis*, 2-7 exons in rice, and 8 exons in soybean. Furthermore, CcAUX/LAXs contained 10 transmembrane helices (**Table 1**, **Supplementary Figure 1**), and the subcellular localization assays indicated that CcLAX1, CcLAX2 and CcLAX4 proteins were localized to the plasma membrane (**Figure 5**), which was consistent with a previous study (Bao et al., 2019). These suggested that the protein structure of CcAUX/LAXs remained virtually unchanged during evolution, possibly due to its functional importance.

### 4.2 Tissue-specific expression analysis of CcAUX/LAX genes

Despite significant gene and protein structural conservation, *CcAUX/LAXs* expression at the transcriptional level varies considerably between tissues/organ (**Figure 4**). Tissue-specific expression profiling indicates that some duplicated gene pairs may play redundant roles in some tissues, such as CcLAX3 and CcLAX7 in roots and CcLAX2 and CcLAX6 in fruits. In *Arabidopsis*, *AtLAX3* regulates lateral root development, and the present study also found that *CcLAX3* and *CcLAX7* gene pairs are highly expressed in roots. It is likely that the *LAX3* gene had retained its original function during evolution (Swarup and Péret, 2012). The role of *PaLAX1* in inflorescence development has been demonstrated, and overexpression of *PaLAX1* in *ataux1* mutants results in the production of multiple inflorescences (Hoyerová et al., 2008). The expression level of *CcLAX1* gene in flowers was higher than in other tissues, indicating that *CcLAX1* might be involved in inflorescence development. Furthermore, most *CcAUX/LAX* genes were expressed at higher levels in roots than in other tissues, while lower expression levels were found in leaves and stems. This may indicate that multiple LAX coordinately participate in the regulation of Chinese hickory root development. Therefore, further detailed analysis of the cell type-specific expression patterns of *CcAUX/LAXs* in different tissues/organ and during different developmental processes will help to identify their specific gene functions.

### 4.3 Expression patterns of CcAUX/LAX genes under salt and drought stresses

Under abiotic stresses such as drought and salinity, plants usually activate various mechanisms to resist the adverse environment (Chai, et al., 2016). Auxin is one of the most essential plant hormones that regulating plant mediating a variety of environmental stress responses (Melnyk et al., 2015; Yu et al., 2017). Recent studies showed that some auxin transporter genes have been involved in abiotic stress responses (Krishnamurthy and Rathinasabapathi, 2013; Rahman, 2013).In *Glycine max*, most *LAX* genes responded to a variety of plant hormone stimulation and abiotic stress (Chai et al., 2016). The majority of *ClLAX*, *ClPIN*, and *ClABCB* genes in stem and root tissues responded to cold, drought, and high salinity (Yu et al., 2017). The majority of *ZmPIN* and *ZmLAX* genes in *Zea mays* were up-regulated in shoots but down-regulated in roots due to drought stress (Yue et al., 2015). In the present study, the expression of *CcLAX1*, *CcLAX6* and *CcLAX7* was up-regulated in the roots of Chinese hickory under mild drought stress, while the expression of other genes had no significantly changed or down-regulated. In addition, the expression of most *CcAUX/LAX* genes were down-regulated at the early stage of salt stress treatment, while the expression of *CcLAX2, CcLAX3* and *CcLAX7* were significantly up-regulated in the roots after 3 days of mild and severe salt stress treatments. Besides, most of the *CcAUX/LAX* expression in stems did not change significantly under drought stress, which was similar in the salt stress treatment. This may be due to the low expression of *CcAUX/LAX* in stems. The expression of CcAUX/LAX after drought and salt stress is irregular, which may be due to the coordinated expression among members involved in auxin uptake or transport, thus regulating the response of Chinese hickory to drought and salt stress.

### 4.4 CcAUX/LAX genes are potentially involved in the grafting healing process

Grafting is a common asexual technique for propagation in Chinese hickory, which can successfully address the issues of tall trees, a long juvenile period, and a low level of improved cultivation. A detailed analysis of morphological characteristics during Chinese hickory grafting revealed that 3, 7, and 14 DAG (days after grafting) are critical time points during the grafting process, showing the development of the necrotic layer, healing tissue proliferation, and differentiation of new vascular tissue in grafts, respectively (Qiu et al., 2016). Based on the transcriptome data of Chinese hickory grafting, auxin-related unigenes were identified as differentially expressed genes (DEGs) during the grafting process (Qiu et al., 2016). Further investigation revealed that the expression of *Aux/IAA*, *GH3*, and *ABCB* family genes changed dramatically during graft healing (Yuan et al., 2018; Xu et al., 2020; Yang et al., 2021). The AUX/LAX transporter is an auxin transporter that aids in the transport of auxin between cells and the formation of vascular bundles. In order to determine the role of *CcAUX/LAX* gene in the grafting process, the expression of *CcAUX/LAX* gene was studied. All *CcAUX/LAX* were in a low expression state in the rootstock, while *CcLAX3* and *CcLAX4/8* were consistently high expression in the scion. This may be because the auxin synthesized in the scion shoots is transported to the grafting healing site by them to promote the connection of vascular tissues, thus promoting the survival of the grafting. During graft healing, auxin plays a role by regulating cell differentiation and vascular bundles. Previous studies revealed that NPA is an auxin transport inhibitor that reduces polar auxin transport (Teale and Palme, 2018). In our study, NPA treatment significantly reduced the expression of several *CcAUX/LAX* genes (such *CcLAX1*, *CcLAX2* and *CcLAX7*) compared to IAA treatment, implying that obstructing auxin transport might impact the AUX/LAX-mediated auxin signaling pathway. However, after NPA treatment, a few genes, including *CcLAX3*, *CcLAX6*, and *CcLAX4/8*, showed increased expression, most likely due to increased expression of some AUX/LAX transporter proteins to maintain auxin homeostasis when auxin transport was inhibited. In contrast, IAA treatment did not increase the expression of *CcAUX/LAX* gene, probably due to the exogenous growth hormone treatment affected the endogenous growth hormone transport. A previous study by our group found that IAA treatment increased while NPA treatment significantly reduced the survival rate of Chinese hickory grafting. Variations in the expressions of the *CcAUX/LAX* genes after IAA and NPA treatment could explain some of our previous findings (Xu et al., 2017). Furthermore, the differential expression patterns of *CcAUX/LAX* family genes suggested that auxin transport during grafting in Chinese hickory might be regulated in a complex manner.

## 5 Conclusion

To investigate the potential roles of auxin influx transporters in response to drought, salt stress and grafting process of Chinese hickory, eight *CcAUX/LAX* genes were identified in Chinese hickory for the first time, which were most closely related to the homologous genes in poplar. CcAUX/LAXs were located on the cell membrane and displayed different expression levels in different tissues, indicating their varying roles during growth and development. Moreover, *cis*-acting elements related to phytohormones and stress responses were detected on the promoters of *CcAUX/LAXs*. The expression levels of *CcAUX/LAXs* were up-regulated or down-regulated to varying degrees during drought and salt stress treatments, indicating their involvement in the response process of plants to abiotic stresses. In addition, the significant changes in the expressions of *CcAUX/LAXs* during grafting and in response to IAA and NPA treatments during grafting may partly explain the mechanism of auxin in regulating Chinese hickory grafting. This study will lay a foundation for further understanding the regulatory mechanisms of auxin transporters during grafting and in response to abiotic stresses in Chinese hickory. In future, further functional analysis and regulatory networks of these genes will be carried out to explore the molecular mechanisms of *CcAUX/LAXs* in response to drought, salt stress and grafting.

## Data availability statement

The original contributions presented in the study are included in the article/**Supplementary Material**. Further inquiries can be directed to the corresponding authors.

## Author contributions

BZ, YH, HY and RW conceived and designed the concept of manuscript. YY, JW, YX, ST, FS and DX performed the experiments. YY, XX, DX, QH, JW and YX analyzed the data. FA, YH, HY, DY and XW did the formal analysis. YY, FA, and HY drafted the manuscript, BZ, FA, XW, AS, LZ, DY, HY, YH, and RW revised and finalized the manuscript. All authors contributed to the article and approved the submitted version.
