# Supplementary material for: Genome-wide identification and expression analysis of AUX/LAX family genes in Chinese hickory (Carya cathayensis Sarg.) Under various abiotic stresses and grafting
Source: Front Plant Sci. 2023 Jan 5;13:1060965. doi: 10.3389/fpls.2022.1060965 (PMC9849883; doi:10.3389/fpls.2022.1060965)
Supplement: Supplementary file 1 [file DataSheet_1.docx]

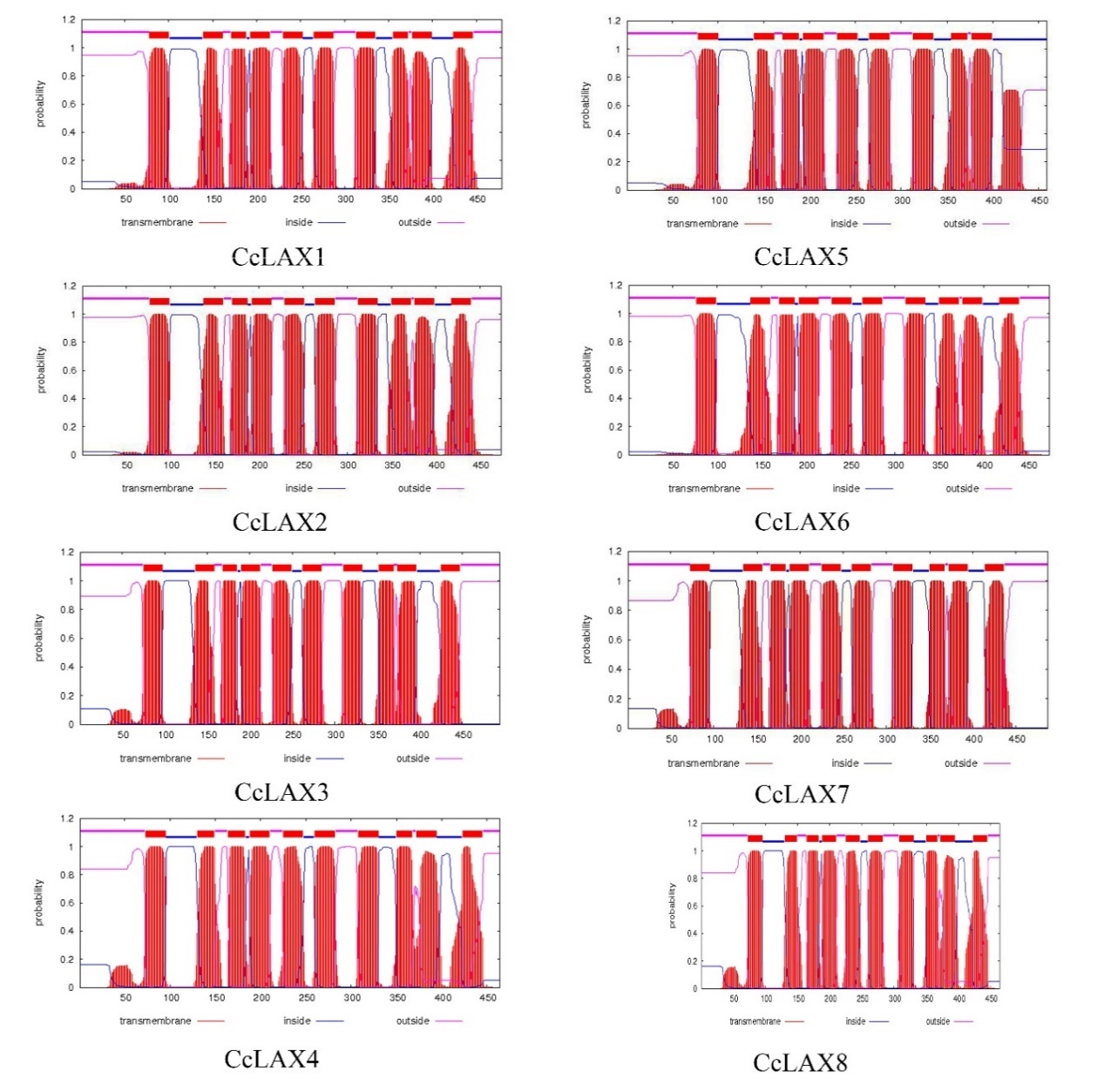


**Supplementary Figure 1.** The predicted transmembrane of CcAUX/LAX poteins, Predict the transmembrane region of the CcAUX/LAX-protein through TMHMM2 software. The predicted transmembrane spiral is shown in red, the area predicted to be on the cytoplasmic side is marked in blue, and the area found outside is marked in pink. (Readers can refer to the online version of this article to understand the reference material about coloring in this illustration.)
