# Supplementary material for: Genome-wide identification and expression analysis of AUX/LAX family genes in Chinese hickory (Carya cathayensis Sarg.) Under various abiotic stresses and grafting
Source: Front Plant Sci. 2023 Jan 5;13:1060965. doi: 10.3389/fpls.2022.1060965 (PMC9849883; doi:10.3389/fpls.2022.1060965)
Supplement: Supplementary file 2 [file DataSheet_2.docx]

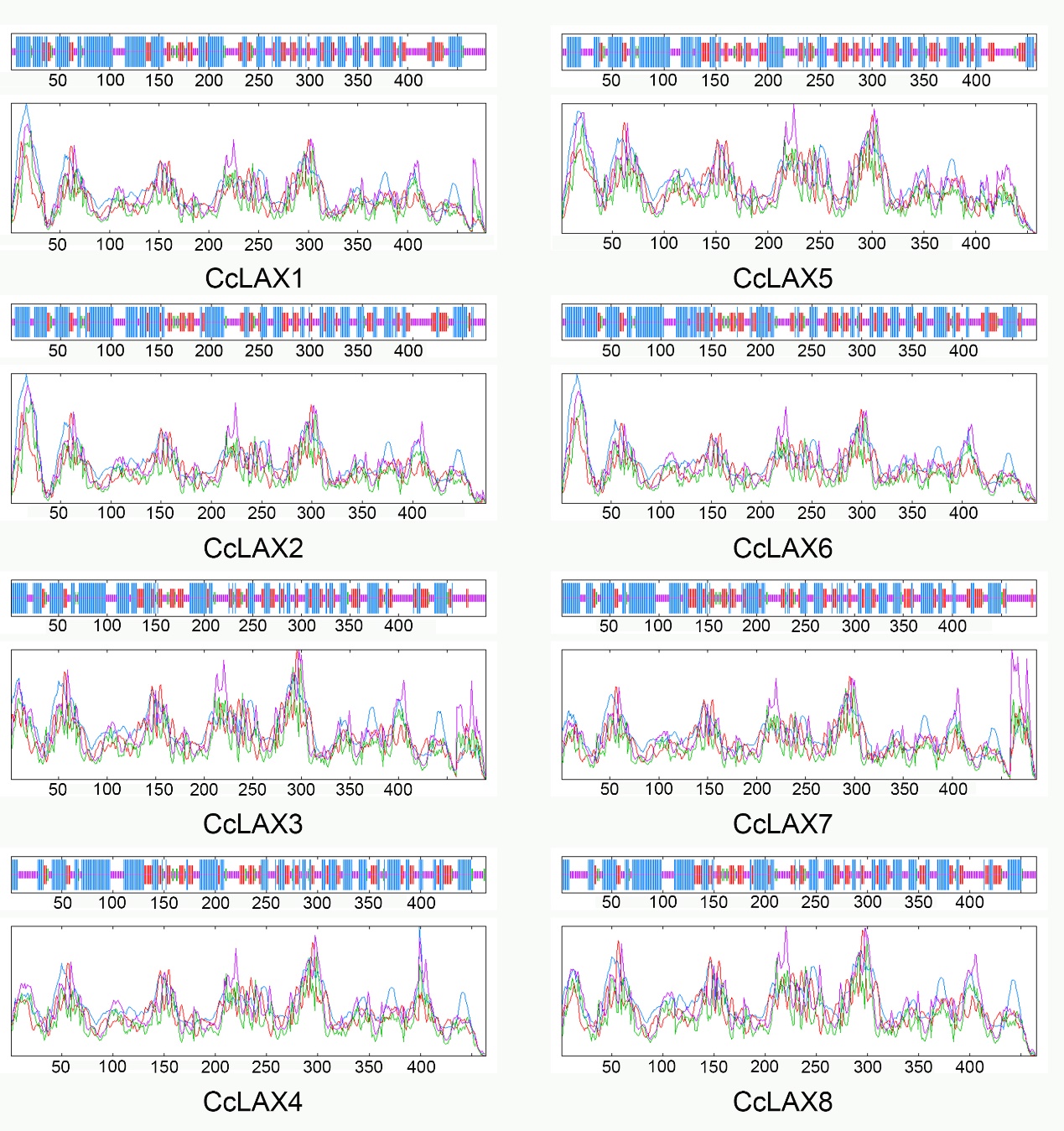


**Supplementary Figure 2.** Secondary structure prediction results for the CcAUX/LAX proteins. The improved self-optimized prediction method (SOPMA) software (http://npsa-pbil.ibcp.fr/cgi-bin/npsa_automat.pl?page=/NPSA/npsa_sopma.html) was used to predict the secondary structure of the CcAUX/LAX proteins. An increased number of extended strands and random coils in the proteins indicate an increased likelihood of the corresponding protein forming an antigenic epitope. Lines in different colors represent different secondary structures: Blue, α helix; green, β turn; red, extended strand; and purple, random coil.
