# Supplementary material for: Genome-wide identification and expression analysis of AUX/LAX family genes in Chinese hickory (Carya cathayensis Sarg.) Under various abiotic stresses and grafting
Source: Front Plant Sci. 2023 Jan 5;13:1060965. doi: 10.3389/fpls.2022.1060965 (PMC9849883; doi:10.3389/fpls.2022.1060965)
Supplement: Supplementary file 3 [file DataSheet_3.docx]

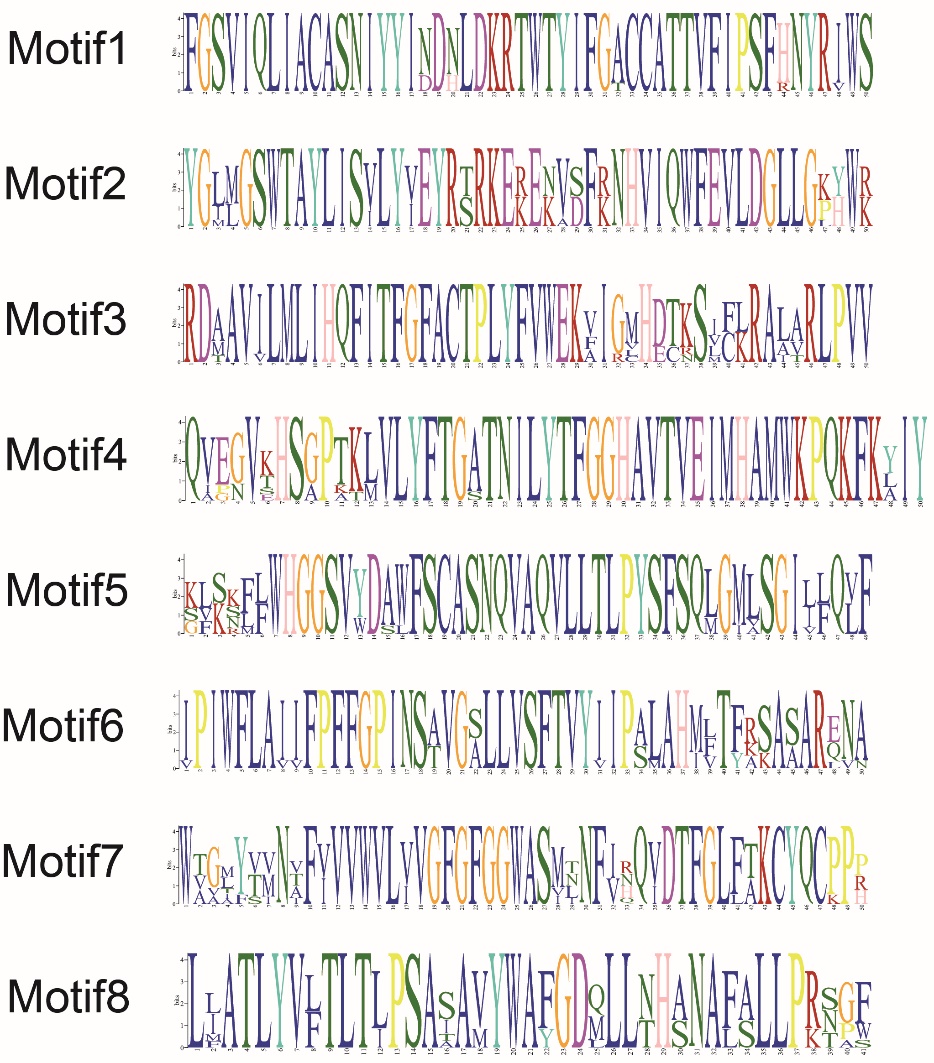


**Supplementary Figure 3.** The conservation of specific amino acids in eight motifs of AUX/LAX proteins.
