# Supplementary material for: Genome-wide identification and expression analysis of AUX/LAX family genes in Chinese hickory (Carya cathayensis Sarg.) Under various abiotic stresses and grafting
Source: Front Plant Sci. 2023 Jan 5;13:1060965. doi: 10.3389/fpls.2022.1060965 (PMC9849883; doi:10.3389/fpls.2022.1060965)
Supplement: Supplementary file 4 [file Table_1.docx]

Table S1 Primer list of *CcAUX/LAX* genes cloning

| **Gene** | **Primer sequence (5’-3’)** |
| --- | --- |
| *CcLAX1* | F:ATGTTGCCTCAGAAGCAAGCAGAGGAAG  R:TCAGTGATGAGGGGCTGCTGCTGCTGGT |
| *CcLAX2* | F:ATGTTACCACAGACACAAGCAGAGGA  R:TTAGTGATTTTTTGCTCCTGGGACTCG |
| *CcLAX3* | F:ATGGCGTCGGATAAGGTGATGGAGA  R:TCAAGGGCTGTGAGGGCGAGGATTGGG |
| *CcLAX5* | F:ATGTTGCCTCAGAGGCAAGCAGAG  R:TTAGTAATGAAATGATGCCAATTTT |
| *CcLAX6* | F:ATGTTACCTCAGAAACAAGCGGAGG  R:TTAGTGATTTTTCGCGGGTGTGGC |
| *CcLAX7* | F:ATGGCATCAGATAAGGTGGTGGAGAC  R:TTAGGGGTGGGGATGGTAGTGAACAG |
| *CcLAX4/8* | F:ATGGCTTCTGAGAAGGTTGAGAC  R:TCAAGCCTTGTGTGGGGGACACTG |
