# Supplementary material for: Genome-wide identification and expression analysis of AUX/LAX family genes in Chinese hickory (Carya cathayensis Sarg.) Under various abiotic stresses and grafting
Source: Front Plant Sci. 2023 Jan 5;13:1060965. doi: 10.3389/fpls.2022.1060965 (PMC9849883; doi:10.3389/fpls.2022.1060965)
Supplement: Supplementary file 5 [file Table_2.docx]

Table S2 The qRT-PCR primer list of *CcAUX/LAX* genes and *CcActin* gene

| **Gene** | **Primer Sequence（5'-3'）** | **Length（bp）** |
| --- | --- | --- |
| *CcLAX1* | F: GCATGTACGTGGTCAATGCT  R: GCTGCTGCTGCTGGTGTTGG | 166 |
| *CcLAX2* | F: CCCAACTGGACTGTCATGTT  R: TGCTCCTGGGACTCGGGGCG | 168 |
| *CcLAX3* | F：CTCACTAGTCACTTCCTCGAAT  R：CTCCATTTCCACATAATTGC | 200 |
| *CcLAX5* | F: CTGCTCGACTGGTAAATAAAAC  R: ATGAAATGATGCCAATTTTATC | 167 |
| *CcLAX6* | F: TCCGAGCTGGACTGCCATTTAT  R: TGATTTTTCGCGGGTGTGGC | 177 |
| *CcLAX7* | F: TACATGGGCCGATGGGTAGG  R: AGATCAAGGCGAGGTGTTGC | 191 |
| *CcLAX4/8* | F: CATTGGTAGGAGGTTGGGC  R: AGCCTTGTGTGGGGGACACTG | 170 |
| *CcActin4* | F:GCTGAACGGGAAATTGTC  R:AGAGATGGCTGGAAGAGG | 188 |
