# Supplementary material for: Genome-wide identification and expression analysis of AUX/LAX family genes in Chinese hickory (Carya cathayensis Sarg.) Under various abiotic stresses and grafting
Source: Front Plant Sci. 2023 Jan 5;13:1060965. doi: 10.3389/fpls.2022.1060965 (PMC9849883; doi:10.3389/fpls.2022.1060965)
Supplement: Supplementary file 7 [file Table_4.docx]

Table S4 Similarity analysis of CcAUX/LAX family protein

|  | CcLAX8 | CcLAX7 | CcLAX6 | CcLAX5 | CcLAX4 | CcLAX3 | CcLAX2 | CcLAX1 |
| --- | --- | --- | --- | --- | --- | --- | --- | --- |
| CcLAX1 | 78.06 | 77.22 | 85.23 | 85.34 | 77.59 | 77.54 | 85.62 |  |
| CcLAX2 | 76.51 | 75.64 | 94.08 | 77.85 | 76.89 | 75.64 |  |  |
| CcLAX3 | 85.56 | 93.98 | 76.33 | 72.57 | 85.53 |  |  |  |
| CcLAX4 | 93.32 | 85.31 | 76.24 | 73.01 |  |  |  |  |
| CcLAX5 | 73.67 | 72.12 | 77.63 |  |  |  |  |  |
| CcLAX6 | 76.51 | 75.91 |  |  |  |  |  |  |
| CcLAX7 | 86.21 |  |  |  |  |  |  |  |
| CcLAX8 |  |  |  |  |  |  |  |  |
